# The brain’s dark transcriptome: Sequencing RNA in distal compartments of neurons and glia

**DOI:** 10.1016/j.conb.2023.102725

**Published:** 2023-05-15

**Authors:** Seth A. Ament, Alexandros Poulopoulos

**Affiliations:** 1 Department of Psychiatry, UM-MIND, and Institute for Genome Sciences, University of Maryland School of Medicine, Baltimore, MD, USA; 2 Department of Pharmacology and UM-MIND, University of Maryland School of Medicine, Baltimore, MD, USA

## Abstract

Transcriptomic approaches are powerful strategies to map the molecular diversity of cells in the brain. Single-cell genomic atlases have now been compiled for entire mammalian brains. However, complementary techniques are only just beginning to map the subcellular transcriptomes from distal cellular compartments. We review single-cell datasets alongside subtranscriptome data from the mammalian brain to explore the development of cellular and subcellular diversity. We discuss how single-cell RNA-seq misses transcripts localized away from cell bodies, which form the ‘dark transcriptome’ of the brain: a collection of subtranscriptomes in dendrites, axons, growth cones, synapses, and endfeet with important roles in brain development and function. Recent advances in subcellular transcriptome sequencing are beginning to reveal these elusive pools of RNA. We outline the success stories to date in uncovering the constituent subtranscriptomes of neurons and glia, as well as present the emerging toolkit that is accelerating the pace of subtranscriptome discovery.

## Introduction

Neural differentiation and nervous system development have long been seen as manifestations of a series of transcriptional programs that endow neuronal and glial subtypes with their distinctive properties. Over the past decade, single-cell and spatial genomics approaches have emerged as powerful tools to comprehensively catalog the cell types and subtypes of the brain. The scalability and cost-effectiveness of single-cell and single-nucleus RNA sequencing (sc/snRNA-seq) allow them to be applied at massive scale in the developing and adult brain across species [1–4].

In a parallel set of methodological breakthroughs, pools of RNA can now be sequenced from distal subcellular compartments of neurons and glia. These approaches have revealed that beyond the RNA in cell bodies sampled by scRNA-seq, distal RNA pools lie in subcellular compartments like axons and dendrites that remain unsampled by scRNA-seq approaches. These unseen pools of compartmentalized subtranscriptomes form what we will call the *dark transcriptome* of the brain (borrowing the term from *dark matter* in astrophysics, which refers to matter in the universe that is not directly detected by current instruments, but we can infer from its gravitational consequences). The dark transcriptome adds an extra layer to the molecular diversity of the brain, one which is more granular than cell-types or subtypes. Molecular diversity at the subcellular level, particularly in compartments of neurons and glia that contribute to synapses, is particularly important for functional diversity, through dynamic local processes in development, plasticity, and regeneration [5–7].

Recently, researchers from the Brain Research through Advancing Innovative Neurotechnologies (BRAIN) Initiative Cell Census Network (BICCN) have achieved the milestones of the first single-cell and spatial genomic atlases for the entire mouse brain [8–10], as well as for >100 regions of the human brain. In parallel, an increasing number of distal subtranscriptomes are being sequenced from selective brain regions in development and adulthood. As such, it is an apt time to take stock of the progress that has been made in documenting the somatic and distal subtranscriptomes of the mammalian brain, the technical advances that are accelerating this field, and considerations of how to integrate subtranscriptome data with cell body data for a full appreciation of the molecular diversity of the developing and adult nervous system.

### The somatic subtranscriptomes of scRNA-seq

To date, the cell body and nucleus are the only two subcellular compartments for which sequencing with single-particle resolution has been achieved in a scalable manner. Some of the largest scRNA-seq genomic datasets for the mammalian brain are being produced by the BICCN, which has used these techniques to sequence the transcriptomes of over 50 million cells in the brains of humans, non-human primates, and mice [11]. Clustering of these cells revealed tens to over 100 transcriptionally distinct cell types in specific brain regions [12–15], with full brain atlases revealing >5000 transcriptionally distinct cell types in total [8,9]. Most of these transcriptional cell types refine established neuronal subclasses defined by known markers, and meta-analyses indicated that they are highly reproducible across multiple datasets and distinct scRNA-seq and snRNA-seq technologies [16]. Often, scRNA-seq has subdivided known neuronal sub-classes into multiple transcriptionally distinct cell clusters, some of which were subsequently validated to have distinct properties within neural circuits [15]. Thus, scRNA-seq has enabled the discovery of hundreds of new neural cell subtypes.

Despite this rapid progress, certain limitations have become clear. In particular, scRNA-seq on its own is insufficient to correlate the transcriptional states of cells to their developmental and physiological states. Thus, while atlases for the transcriptionally distinct cell types in many adult brain regions are now available, our understanding for the dynamic changes within these cell types during development and plasticity is often rudimentary [17–19]. The challenge is not merely to generate sufficient scRNA-seq data spanning these conditions. More fundamentally, transcriptome sequencing alone does not enable matching of transcriptional signatures to corresponding cellular processes such as axon outgrowth and synaptogenesis. As discussed below, we believe the full appreciation of the molecular diversity underlying development and plasticity is lacking until the corresponding distal subtranscriptomes of axons, dendrites, glial processes, and synapses fill in the missing dark transcriptome in the scRNA-seq record.

An additional challenge is to better understand the extent to which transcriptomic cell types mirror the morphological and functional characteristics of neurons. Multimodal technologies to co-assay a cell’s transcriptome along with its morphology and physiological characteristics (e.g., Patch-seq) made it possible in some cases to determine the extent to which these transcriptional cell clusters predict anatomical and functional variation. In one of the largest Patch-seq experiments to date, ‘trimodal’ Patch-seq was used to measure the transcriptomic, morphologic, and electrophysiological features of over 1300 neurons in adult mouse primary motor cortex [20]. Reassuringly, in many cases, transcriptomic clusters corresponded to neuronal populations with specific morphological and physiological properties. However, at finer levels of clustering, many adjacent transcriptomic subtypes displayed indistinguishable anatomical and physiological features. Moreover, these phenotypes varied continuously from one transcriptomic cell type to another. Thus, a cell’s scRNA-seq profile does not always predict its properties, suggesting that unseen transcripts may contribute to morphology and function, and thus highlighting an underappreciated role for the dark transcriptome.

One factor contributing to the disconnect between transcriptomic and functional states of neurons is that the cellular transcriptomes assayed by droplet-based sc/snRNA-seq represent biased subtranscriptomes rather than a representation of the complete transcriptome of the cell. This is perhaps obvious for snRNA-seq, as transcripts present in the nucleus typically represent less than 10% of mRNA. In contrast, data from scRNA-seq experiments are often referred to as ‘whole cell’ transcriptomes. Yet scRNA-seq is more accurately described as profiling the subtranscriptome of the cell body (also known as soma or perikaryon). scRNA-seq of brain tissue universally requires a step to dissociate cells from brain parenchyma, during which most neurons and glia lose their processes. As such, single-cell approaches do not see this fraction of the transcriptome that is lost with the distal processes. We estimate this dark transcriptome to represent over 40% of the total transcriptome in an adult mouse brain (Box 1).

In subsequent sections of this review, we will discuss how new subcellular transcriptomic approaches to sequence the subtranscriptomes of neurons and glia could aid in understanding the relationships between a cell’s transcriptomic states and its developmental, morphological, and physiological characteristics.

### Distal subtranscriptomes in neurons

**Figure 1:**
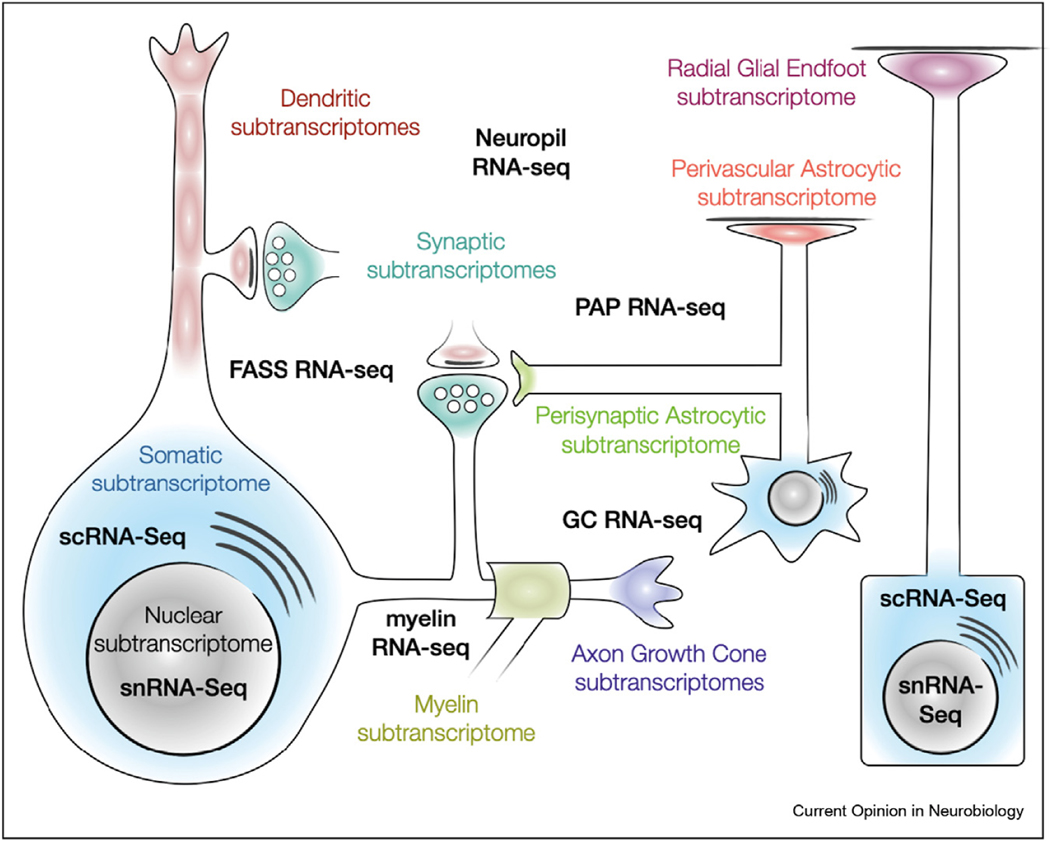
Schema of the subtranscriptomes of the mammalian brain.

The neuropil of the brain is rich in RNA that resides in distal processes of neurons and glia (Figure 1). These sets of RNAs, born in the nucleus, actively travel long cytoplasmic distances from the soma through neural processes to their destined subcellular sites, which can be macroscopically distant from their parent cell body. Much is known from in vitro studies on the biology of RNA transport [21,22], the mechanisms extending transcript half-lives to allow for the long journey [7,23], and the regulation of local translation [6,24]. While a variety of ingenious cell-culture methods based on filtration barriers and microdissections have yielded abundant data on subcellular RNA in vitro [25], here we focus on experiments documenting subtranscriptomes in the mammalian brain with anatomical and cell-type specificity (Table 1). This will help us consider how distal subtranscriptome data can complement scRNA-seq data and how together they may illuminate a hidden aspect of molecular diversity in the brain.

Distal subtranscriptomes in neurons have slowly been emerging over decades. The first RNA localization studies focused on selected gene products with in situ hybridization. These hypothesis-driven experiments confirmed the presence of distally localized transcripts and pioneered subcellular transcriptomics [26]. Today, the field of neural subtranscriptomics seeks ever higher sequencing depths of subtranscriptomes with increasing subtype specificity in ever more native contexts. Indeed, it is the technical difficulties behind obtaining pure subtype specific subcellular transcripts in vivo that have confined the field to slow progress with a trickle of heroic efforts documenting yet one more subtranscriptome. This is in stark contrast to the torrent of progress made in the scRNA-seq world, where transcriptomic datasets are produced faster than we can consume—or interpret.

After the unequivocal demonstration by Os Steward and William Levy of mRNA and ribosomes forming polysomes in neuropil and dendritic spines [27], early in vivo studies took advantage of cell types in the brain with opportune stratification, like Purkinje cells or hippocampal pyramidal cells, to localize specific transcripts to layers with dendrites and axon initial segments [28]. The detection of local RNA in proximity to synapses in dendrites coincided with the appreciation that local protein synthesis is required for long-term plasticity and learning [29]. These findings led to the notion of activity-dependent postsynaptic local translation being critical for plasticity and learning [30].

From the single-cell in situs of the 1990s, the field is now using spatial transcriptomic methods like multiplexed error robust FISH (MERFISH) and fluorescent in situ RNA sequencing (FISSEQ) with subcellular resolution to reveal cell-wide subtranscriptome distributions at the synapse level [31,32]. While achieving this at subcellular resolution in dense neuropil in vivo remains a big challenge, a combination of expansion microscopy with in situ sequencing, termed ExSeq [33], increased the resolution of FISSEQ [34] to sequence individual pyramidal neuron cell bodies and proximal dendrites in the intact hippocampus, producing the first subtranscriptome-resolved in situ sequencing of neurons in the brain. Until these technologies are brought to scale and achieve sampling depth and resolution adequate for the distal subtranscriptomes, RNA-seq on subcellular fractions from the brain currently provide the mainstay of in vivo subtranscriptome datasets.

### Neuropil and dendritic subtranscriptomes

One of the first RNA-seq studies to probe the distal subtranscriptome in vivo was by Cajigas et al., taking advantage of hippocampal stratification to microdissect *stratum pyramidale* to sequence the somatic subtranscriptome, and *stratum radiatum* and *stratum lacunosum moleculare* to sequence neuropil, enriched for the dendritic subtranscriptomes of CA1 pyramidal neurons [35]. While neuropil fractions contain several components other than pyramidal neuron dendrites, including glia, interneurons, and axons with presynaptic terminals, this landmark study showed the way forward in applying RNA-seq approaches to distal subtranscriptomes in specific circuits in vivo.

Refinements of the neuropil microdissection and RNA-seq approach have been fruitful in understanding how subtranscriptomes contribute to the molecular diversity along the topography of hippocampal subfields and strata. A follow-up study looking at transcript isoforms in soma and neuropil found large divergence in 3′ untranslated regions (UTRs), giving neuropil-enriched isoforms longer half-lives [36]. Further examination of transcript isoforms found that neuropil subtranscriptomes are divergent across the different hippocampal subfields, with the neuropil subtranscriptome of CA2 being enriched in transcripts related to mitochondria, while CA1 and CA3 neuropil subtranscriptomes were not [37].

Analyzing published datasets, Ha et al. mined neuropil subtranscriptomes [35] together with soma subtranscriptome data of microdissected and manually sorted cell bodies from the hippocampal subfields [38]. Their approach revealed that alternative splicing and alternative polyadenylation critically contribute to diversification even within cell subtypes across the axes of the hippocampal formation [39]. Interestingly, they identified an axis of variability along the hippocampal formation that was characterized by variability in the 3′ UTR of transcript isoforms that often determine localization and half-life [22]. Intersecting these datasets revealed high correspondence of the topographically variable transcripts to those enriched in the neuropil subtranscriptome. Specifically, both transcript sets were enriched in ribosomal protein mRNAs (rpmRNAs), a class that has consistently been found enriched in distal subtranscriptomes (see below). This study further exemplifies the great potential for comparatively integrating existing scRNA-seq datasets of soma with distal subtranscriptomes to extract further aspects of molecular diversity with respect to local circuits.

An alternative approach to sample dendritic subtranscriptomes in vivo with cell-type specificity has been to sequence locally translated RNAs via translating ribosome affinity purification approaches, RiboTag, and Translating Ribosome Affinity Purification (TRAP) [40–42], which we collectively term here RiboTRAP. These approaches use conditional or viral tagging of ribosomes, enabling purification of mRNAs that are attached to ribosomes, often in the process of being translated. The intersection of cell type-specific lines or viruses and anatomical separation of cell body, dendritic, and axon projection fields has produced datasets of in vivo subcellular translatomes with high specificity. One caveat to riboTRAP is that only ribosome-bound transcripts are sampled. Thus, these approaches miss subtranscriptomes that are dormant in other ribonucleoprotein particles, or which undergo regulated non-constitutive bouts of translation. On the other hand, these methods can be used to reveal such translational dynamics serendipitously or by design.

RiboTRAP approaches have been combined with microdissection to isolate the dendritic translatomes in hippocampal neuropil [43,44] and cerebellum [45], allowing a higher purity dendritic translatome devoid of from glial and interneuron contributions in neuropil. Intersectional cell-type specific RiboTRAP labeling has been combined with subcellar fractionation to enrich for neuropil transcripts from corticofugal projection neurons, cortical GABAergic interneurons [46], midbrain dopaminergic neurons [47], and brainstem serotonergic neurons [48]. In hippocampus and midbrain, these studies determined pervasive translation of the dendritic subtranscriptomes.

The team of Peter Scheiffele applied riboTRAP at large scale across rodent forebrain to document transcript isoform diversity. They found that even closely related neuron subtypes in distinct anatomical subregions display divergence in transcript isoforms through alternative splicing and transcript maturation [49]. Different stages of RNA maturation appear to characterize distinct subtranscriptomes in cultured cortical neurons [50]. Alternative polyadenylation sites that determine 3′ UTRs represent one fifth of all transcript isoform diversity in the forebrain [49]. These findings indicate that alternate RNA isoforms [37] and maturation contribute to subtranscriptome diversity.

Taking a highly multiplexed in situ hybridization approach in cultured hippocampal neurons, Zhuang et al. probed 4700 mRNA species with MERFISH and expansion microscopy and identified over 400 transcripts specifically enriched in dendrites [51]. One striking finding comes from their ability to quantify copy numbers of the RNA species in each compartment. The copy number for any given RNA molecule in the dendritic subtranscriptome is far less than the number of synapses per neuron, indicating that there is no standard subtranscriptome module present at each synaptic site. These spatial transcriptomic data rather point to more heterogeneous clusters in the dendritic subtranscriptome with sparse foci along different dendritic segments, with interesting implications for hypothesis involving synapse-specific local translation in long-term plasticity [52,53].

### Growth cone subtranscriptomes in development and regeneration

Growth cones, specialized structures at the leading edges of growing processes, were one of the first distal structures shown to harbor their own subtranscriptomes [54]. Early transcriptome-scale studies manually isolated growth cones from cultured hippocampal neurons [55], leading to the identification of the first targeting sequences in mRNA localizing to growth cones [21]. These studies coincided with evidence for local translation playing a role in axon guidance during development [56,57] and axon regrowth during regeneration [58].

Studies in culture were followed by in vivo evidence of local translation and the first growth cone subtranscriptomes sequenced in vivo. By labeling ribosomes in the retina and purifying ribotagged transcripts along their projection route, Shigeoka et al. sequenced the translatome from developing and adult retinotectal axons, providing some of the first projection-specific axon translatomes in vivo [59].

**Figure 2:**
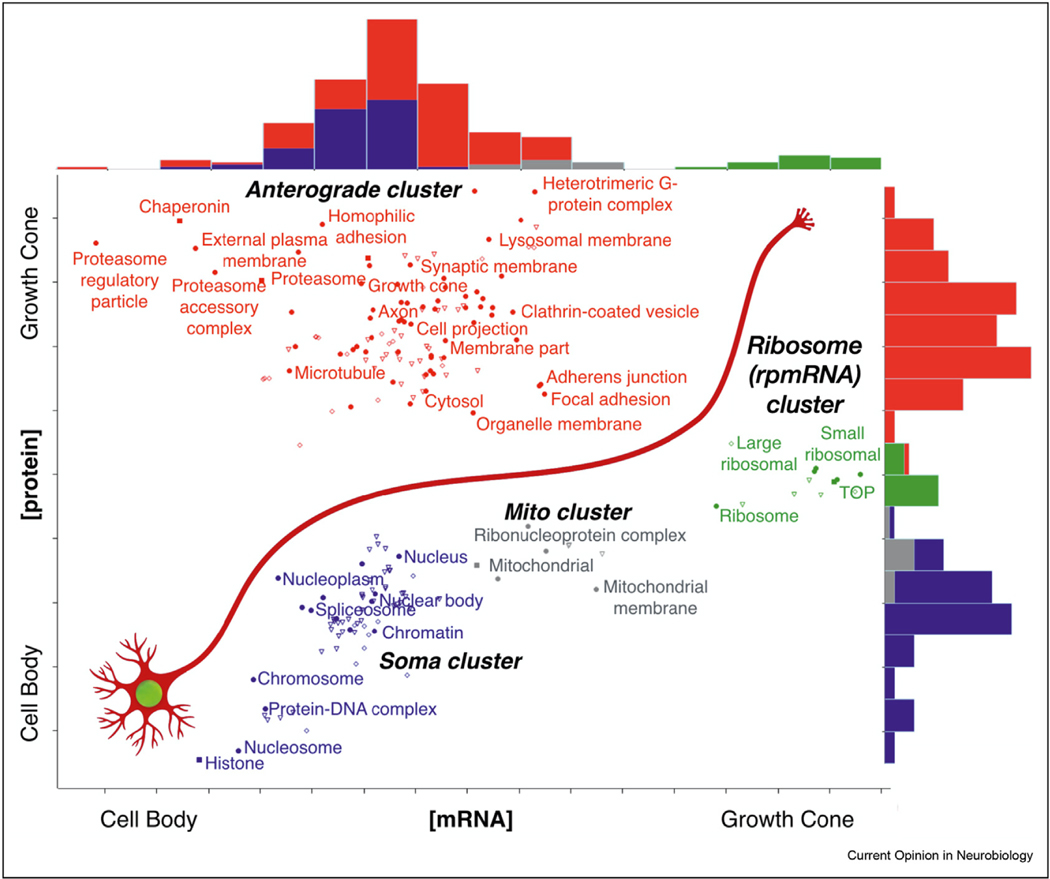
Categories of mRNA transcripts and protein products in soma versus axon growth cone of a cortical projection neuron in the developing brain. Subtranscriptome to subproteome enrichment cross-correlation is 0.014 across the dataset, indicating significant non-local functions for subtranscriptomes. Adapted from the study by Poulopoulos et al. [60].

To sequence subtranscriptomes from growth cones of a specific axon projection from a single neuron subtype in vivo, we developed a growth cone sorting and RNA-seq approach. This combines cell type-specific fluorescence labeling and subcellular fractionation to obtain growth cones from a projection target area, with small-particle sorting to collect labeled growth cones for RNA-seq and proteomics. With this approach, we sequenced the axon growth cone subtranscriptome of an intracortical projection in the early postnatal mouse brain [60,61]. We quantified subcellular enrichments by comparing sorted parent cell bodies from the projection source to sorted growth cones from the projection target area. This enabled plotting the data as gene-product enrichment vectors showing RNA and protein enrichments between soma and growth cone compartments (Figure 2).

### The presynaptic subtranscriptome

Presynaptic terminals were thought, until recently, devoid of translation machinery. The historical paucity of evidence for presynaptic mRNA and ribosomes in the adult brain stood in contrast to the abundant evidence for axon growth cone mRNA and local translation in the developing brain. A series of recent subtranscriptomic and local translatome studies in adult rodents have conclusively shown that presynaptic terminals do contain subtranscriptomes and that local axon translation occurs in the adult, albeit scarcely.

RiboTRAP methods from anatomically isolated axon fields of labeled projection neurons provided the first sequencing of axon translatomes in the adult brain. Shigeoka et al. applied riboTRAP to retinal ganglion cells and identified translating transcripts in the adult mouse tectum [59]. Ostroff et al. applied viral riboTRAP in adult rats to sequence the translatome of cortico-amygdalar projections [62].

The question of whether axon local translation occurs at the presynaptic terminal was directly addressed by Hafner et al. using an approach that paired Fluorescence Activated Synaptosome Sorting (FASS) [63] with RNA-seq to obtain the first sequenced presynaptic subtranscriptomes from glutamatergic terminals in the forebrain [64]. These studies unequivocally show the presence of a presynaptic subtranscriptome and its local translation in the adult brain.

However, in quantitative terms, the presynaptic compartment translates very little and contains very few transcripts. Comparing axon translatomes across three developing stages and in the adult, Shigeoka et al. found that only a quarter of transcripts were represented in all stages and that the intensity of axon translation tapers off significantly as the brain matures [59]. Applying riboTRAP and FASS RNA-seq on dopaminergic axon terminals in the striatum, Hodson et al. found no axon translatome or subtranscriptome signal above background [47]. By quantifying background, they imputed an upper range of RNA molecules per dopaminergic synaptosome between 0.2 and 2 mRNA molecules, which concurs with estimates in glutamatergic synaptosomes from cortex [64]. Similarly low numbers were quantified by single-molecule in situ sequencing in cultured neurons, where only 4% of detected RNA species enriched in axons, and that over 95% of transcripts are represented on average by less than a single copy per axon [51].

Taken together, these numbers suggest that axons and presynaptic terminals in mature neurons will not all have a standard axon subtranscriptome. Indeed, having a subtranscriptome at all appears to be a scarce phenomenon for any given synapse in the adult brain. Together with the evidence that presynaptic subtranscriptomes exist and are translated in the adult brain, these data point to an interesting layer of diversity at synapses: some will have and some will not have presynaptic subtranscriptomes. Given the robust evidence that plasticity and learning require local translation [25,65], the presence of a translation-competent subtranscriptome at select axon terminals may function as an intersectional filter for which synapses can—and which synapses cannot—undergo translation-dependent long-term plasticity [53].

### The distal subtranscriptomes of glia

Distal processes of non-neuronal cells in the neuroglial lineage have also provided abundant evidence of distal subtranscriptomes. Neural progenitors have subtranscriptomes and robust translation machinery in radial glial endfeet [66]. RNA can be sequenced from myelin formed by oligodendrocyte processes [67], with myelin basic protein mRNA being one of the early transcripts known to have functionally critical enrichment in subcellular fractions [68]. Local subtranscriptomes are also present in peripheral astrocytic processes surrounding blood vessels (perivascular astrocytic subtranscriptome) [69] and synapses (perisynaptic astrocytic subtranscriptome) [70]. Neuronal and glial subtranscriptomes (Figure 1) have partially overlapping transcript groups [71]. However, the paucity of subtranscriptome sampling across different cell types and developmental stages prevents us from having a full picture of how the subtranscriptome commonalities and specificities relate to biological events rather than to experimental particulars.

To isolate the subtranscriptome of astrocyte endfeet that surround blood vessels and form an important component of the blood—brain barrier, the team of Martine Cohen-Salmon came up with a clever preparation to sequence the perivascular astrocyte endfoot subtranscriptome in vivo. Mechanically isolated brain vessels retain perivascular astrocyte endfeet attached to basal lamina, while astrocyte cell bodies and other neuronal cells and processes are lost in the preparation. Subsequent partial enzymatic digestion removes astrocyte endfeet RNA, while vascular RNA remains protected by the basal lamina. Sequencing these two preparations and comparing what was lost in the digestion step reveals the perivascular astrocytic subtranscriptome. Applying astrocyte-specific riboTRAP to the brain vascular prep additionally revealed the perivascular astrocytic translatome [69].

Applying the astrocyte-specific riboTRAP approach to synaptosome fractions produced the perisynaptic astrocytic translatome of the tripartite synapse from cortex [70] and hippocampus [72]. These pioneering studies demonstrated that perisynaptic astrocytic subtranscriptomes contain transcript isoforms with distinct 3′ ends [70], as seen previously in neuron distal subtranscriptomes. Interestingly, the persynaptic astrocytic translatome was seen to dynamically respond to synaptic activity [73] and to learning [72]. Further investigation into this nascent field is sure to yield new biology at the synapse.

### Non-local functions of subtranscriptomes

Undoubtedly, subtranscriptomes provide a pool of localized mRNAs for local translation that serves the replenishment of the local proteome [7]. To this end, transcripts encoding synaptic proteins are found in the synaptic subtranscriptome [64], cytoskeletal transcripts are found in growth cones [5], and transmitter-metabolizing enzymes are translated in perisynaptic astrocytic processes [70]. However, there are some consistent enrichments found in subtranscriptomes that do not correspond to equivalent enrichments in the local proteome. In fact, when we measured the cross-correlation between subtranscriptomes and subproteomes in soma and axon growth cones, we found it to be near zero (0.014) [60] (Figure 2), suggesting a significant non-local component for its function.

Non-local functions of the nuclear and somatic subtranscriptomes are obvious. For example, many transcripts in the rough endoplasmic reticulum will have non-local functions by producing proteins that are transported to distal neuronal processes (represented by the ‘anterograde cluster’ in Figure 2). Non-local functions of distal subtranscriptomes, however, have been more difficult to pinpoint.

Evidence for non-local function comes from one of the most consistent findings in distal subtranscriptomes across cell types, which is also the most peculiar to interpret from the perspective of local translation serving the local proteome. The most consistently enriched transcripts in distal subtranscriptomes are rpmRNAs, briefly mentioned above. These transcripts produce the protein components of ribosomes and are necessary for ribosome biogenesis in the nucleus.

Even though ribosomes are present in distal foci where local translation occurs, they are by far a minor fraction of local protein, compared to the 5–20% of the total cellular proteome [74]. A characteristic example is the presynaptic terminal, which was thought to be devoid of ribosomes altogether due to their small number [64,75]. In all studies of the presynaptic subtranscriptome and translatome, rpmRNAs that produce ribosomal proteins are among the most enriched transcripts [47,59,62,64], despite the very few ribosomes at the presynapse. In axon growth cones, rpmRNAs represent over 80% of the transcripts enriched in growth cones, while ribosomal protein was de-enriched [60] (Figure 2). Significant enrichment of rpmRNAs is also observed in radial glial endfeet [66], as well as in multiple neuron culture studies [76]. rpmRNAs are even found enriched at apical and leading edge subtranscriptomes of epithelial cells, functioning to regulate ribosome biogenesis [77,78].

The high enrichment of rpmRNAs in distal subtranscriptomes has been difficult to interpret according to the model of local mRNA serving as a proximal source for the local proteome [25]. A local-transcripts-for-local-proteins interpretation has been postulated for rpmRNAs involving in situ ribosome repair and remodeling, where locally synthesized ribosomal protein from subtranscriptome rpmRNAs is incorporated into existing local ribosomes. This model was recently demonstrated by two studies showing that refurbishment of superficial ribosomal proteins occurs through local translation independent of the nucleus [79,80], with interesting ramifications for ribosome heterogeneity [81].

An alternative model is that growing processes, such as growth cones, supply a significant amount of ribosomal protein for the rest of the cell. We have suggested that a potential benefit for such circuitous trafficking of RNA and protein may be regulation of cell-wide ribosomal content by the local conditions at sites of most intense growth [60], proposed in neurons as the mTOR outpost model [53]. It is supported by the finding that transcripts for most—if not all—ribosomal proteins are enriched in leading processes, not just those available for local replacement on the surface of ribosomes [6,25,76].

The notion of non-local functions of distal subtranscriptomes opens a range of interesting implications for cross-compartment regulation, in which local subtranscriptomes at select subcellular sites, such as synapses, influence aspects of cell biology in distant parts of the neuron, such as the nucleus. Further investigations into non-coding RNAs and coding-independent functions of mRNAs [82] in local subtranscriptomes are also likely to yield new and unexpected biology. Understanding local and non-local functions of distal subtranscriptomes will be a key aspect of future research.

## Conclusions and prospects

Distal subtranscriptome datasets from in vivo sources are only available for a limited set of samples. This is in contrast to cell body subtranscriptome datasets that through scRNA-seq scaling are approaching full coverage of cell types, brain areas, and developmental time-points. A major hindrance to distal subtranscriptome research in vivo has been the lack of scalability of methods to extract subcellular RNA with cell-type and compartment specificity that are generalizable across the brain. Indeed, the distal subtranscriptome is missing its equivalent transformative technology that has enabled sequencing of the somatic and nuclear subtranscriptomes at massive scales.

With that in mind, there are emerging technologies that have potential for becoming the transformative scalable methods for subtranscriptome research. Sub-cellular resolution spatially resolved transcriptomics holds great promise. The advantage over subcellular fraction RNA-seq methods is contiguous transcript identification along cell compartments without the binning of fractionation approaches. To date, most sub-cellular resolution spatial transcriptomics studies have utilized highly multiplexed in situ hybridization, as well as in situ sequencing approaches. Typically, these technologies are targeted to hundreds or a few thousands of transcripts. Increasing the plexity of these technologies toward an unbiased representation of the transcriptome is primarily limited by optical crowding. Thus, combining in situ sequencing with expansion microscopy is a compelling strategy to achieve an unbiased representation [33]. There is a need for technological improvement to increase sampling depth and resolution in distal compartments. The current best is in vivo sequencing limited to proximal dendrites within 100 μm from the cell body with an average of 30 reads per neuron. There is a long way to go, but as sampling and resolutions increase, spatial transcriptomics will become a staple for studying the molecular diversity of subtranscriptomes across brain circuits.

Proximity labeling approaches, including Halo-seq [83] and Chromophore-Assisted Proximity labeling and sequencing (CAP-seq) [84], also hold potential for in vivo subtranscriptome research. In principle, these technologies can be applied analogously to the recent uses of proximity labeling of subproteomes with cell-type and subcellular specificity in vivo [85]. Such approaches have the potential to offer versatile ways to increase the granularity of subtranscriptomes sampled. Labeling enzymes fused to protein markers that define subcellular compartments, such as synaptic machinery, or RNA-containing structures like RNA granules or mTOR outposts, will enable sequencing of ever more local and more specialized subtranscriptomes, allowing us to move from describing subtranscriptomes of broad compartments, to describing focal subtranscriptomes of specialized structures.

Our understanding of the diversity and dynamics of subtranscriptomes in the brain will be critical to our understanding of how local molecular diversity contributes to the development and plasticity of complex circuitry. Systematic documentation of the dark transcriptome in neurons and glia will critically fill in blind spots in our appreciation of cellular diversity left by soma-only scRNA-seq approaches. A fuller understanding of distal subtranscriptomes may be transformative to the interpretation of datasets using bulk RNA-seq to assay entire brain regions, which have been applied at scale to study differences in the brains of donors who died with neurological or neuropsychiatric disorders. Samples of brain tissue will contain mixes of somatic and distal subtranscriptomes, the latter including transcripts from distant cells projecting their subtranscriptomes into the sample area. Reinterpretation of transcriptomic readouts from this lens may yield new findings that are buried within the dark transcriptome of the brain.

## Figures and Tables

**Table 1:** Methods to sequence subtranscriptomes from mammalian brain in vivo.

Subtranscriptomes	Target Dataset	Method	Reference	Protocol reference	Specificity
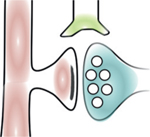	**Neuropil subtranscriptomes**	Microdissection of neuropil strata and RNA-seq	Cajigas et al., 2012 Tushev et al., 2018		Microdissection of CA1 *s. radiatum* and *s. lacunosum moleculare*
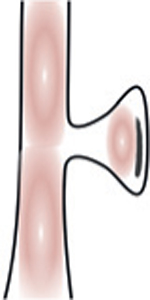	**Dendritic translatome**	Microdissection RiboTRAP	Kratz et al., 2014 Ainsley et al., 2014 Glock et al., 2021, Hobson et al., 2022		Conditional or viral riboTRAP, synaptosome prep or microdissection to deplete somatic
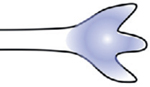	**Axon Growth Cone translatome**	Axon RiboTRAP	Shigeoka et al., 2016	Shigeoka et al., 2018	Retinotectal projections at three developmental stages
	**Axon Growth Cone subtranscriptome**	Growth Cone Sorting RNA-seq	Poulopoulos et al., 2019	Engmann et ai., 2022	Transcallosal axon growth cones from layer II/III cortical neurons
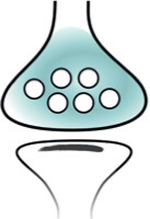	**Presynaptic translatome**	RiboTRAP	Shigeoka et al., 2016 Ostroff et al., 2019 Hobson et al., 2022	Shigeoka et al., 2018	TE3-LA cortico-amygdalar projection. In rats by AAV, no genetic strain required.(?)
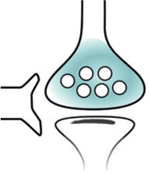	**Synaptic subtranscriptome**	Synaptosome Sorting (FASS) RNA-seq	Hafner et al., 2019		Synaptosomes positive for vGiutl from forebrain.
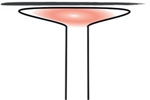	**Perivascular Astrocytic subtranscriptome**	Vascular prep and partial digestion RNA-seq	Boulay et al., 2017	Boulay et al., 2019	Brain-wide vascular prep retains astrocyte endfeet.
	**Perivascular Astrocytic translatome**	Astrocyte riboTRAP on vascular prep	Boulay et al., 2017	Boulay et al., 2019	
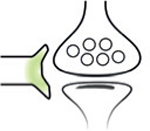	**Perisynaptic Astrocytic translatome**	Astrocyte riboTRAP on synaptosome	Sakers et al., 2017 Mazaré et al., 2020		Synaptosome fraction astrocyte riboTRAP in cortex and dorsal hippocampus
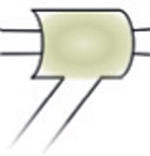	**Myelin subtranscriptome**	Myelin prep RNA-seq	Thakurela et al., 2016		RNA-seq from biochemically purified myelin fraction
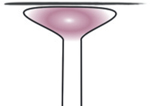	**Radial Glial Endfoot subtranscriptome**	Meningeal prep Radial Glial RBP-IP	Pilaz et al., 2016		Use of RNA-binding protein to immunoprecipitate endfoot RNA from meningial prep
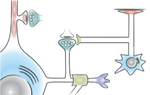	**In situ sequenced subtranscriptomes**	ExSeq (FISSEQ + expansion microscopy	Alon et al., 2021		Expansion microscopy for in situ sequencing with subcellular resolution

## Data Availability

No data was used for the research described in the article.

## Box 1. How much dark RNA is in the brain?

In analytical data from human embryonic and postnatal brains, we can see the brain’s RNA content continues to grow well after cell proliferation growth has ended, indicated by the plateau in DNA content (graph adapted from the study by Winick [86]). This corresponds to RNA content increasing during neuropil growth as cells extend processes in early postnatal development. How much of the brain’s RNA ends up in distal processes invisible to scRNA-seq methods? In other words, how much dark transcriptome does the brain contain?

To get an estimate, we used measurements by the Allen Institute [87] of the amounts of RNA in dissociated cell bodies by cell type. This RNA corresponds to the visible transcriptome that is sequenced by sc methods. We calculated the number of each cell type in the brain using data from the Allen Mouse Brain Atlas analyzed by the Blue Brain Cell Atlas [88] (portal.bluebrain.epfl.ch) to find the proportions of each cell-type in the brain. These proportions are largely consistent with recently released spatial transcriptomic datasets (braincelldata.org) from the BICCN [8,9]. We quantified cell numbers by applying these proportions to the measurement of 97.7 million total cells in an average 8-week old C57BL/6 mouse brain by the CUBIC Atlas v. 1.2 (cubic-atlas.riken.jp) [89,90]. Multiplying the number of cells to the RNA per cell body for each cell type, we calculated that an 8-week old C57BL/6 mouse brain contains 812 ng of RNA within cell bodies. Estimating that approximately 5% of that corresponds to mRNA, this would make the visible mRNA transcriptome of the brain to about 40 ng.

The total amount of RNA in the brain of an 8-week old C57BL/6 mouse, the same age and strain as the Allen Institute and CUBIC-Atlas analyses, was measured at 1.38 ± 0.05 mg (down from a peak of 1.83 mg RNA content at 2 weeks after birth) [86]. Subtracting the 812 ng of visible RNA, we calculate that 568 ng—more than 40% of total RNA—is inaccessible to scRNA-seq methods, corresponding to the dark transcriptome. Indeed, this is an underestimate, as neuropile fractions are ribosome- and rRNA-poor compared to whole brain fractions [60,80]. This means that of the 568 ng of total neuropil RNA, a smaller fraction is going to be rRNA, and thus a larger fraction than 5% will correspond to mRNA, raising the proportion of coding dark transcriptome to potentially well over 40%.

**Table:**

Cell types	Excitatory neurons	Inhibitory neurons	Oligo-dendrocytes	Astrocytes	Microglia	Endothelial cells [4]	Total [90]
Cell numbers in C57BI76 8-week old mouse brain [88]	46,870,000	11,918,000	15,347,000	4,388,000	8,107,000	11,069,000	**97,699,000**
RNA per cell body (pg) [87]	12.2	10.8	3.1	2.5	1.6	2.4	
Total cell body RNA (μg)	571.8	128.7	47.6	11.0	13.0	26.6	**799**
Total RNA in C57BI76 8-week old mouse brain (μg)							**1380**
Calculated dark RNA (μg)							**581**
Dark transcriptome % (lower bound)							**42%**
